# A Homogenous Fluorescence Quenching Based Assay for Specific and Sensitive Detection of Influenza Virus A Hemagglutinin Antigen

**DOI:** 10.3390/s150408852

**Published:** 2015-04-15

**Authors:** Longyan Chen, Suresh Neethirajan

**Affiliations:** BioNano Laboratory, School of Engineering, University of Guelph, Guelph, ON N1G 2W1, Canada; E-Mail: lchen266@uwo.ca

**Keywords:** influenza virus, hemagglutinin, glycan, quantum dot, gold nanoparticle, Förster resonance energy transfer

## Abstract

Influenza pandemics cause millions of deaths worldwide. Effective surveillance is required to prevent their spread and facilitate the development of appropriate vaccines. In this study, we report the fabrication of a homogenous fluorescence-quenching-based assay for specific and sensitive detection of influenza virus surface antigen hemagglutinins (HAs). The core of the assay is composed of two nanoprobes namely the glycan-conjugated highly luminescent quantum dots (Gly-QDs), and the HA-specific antibody-modified gold nanoparticle (Ab-Au NPs). When exposed to strain-specific HA, a binding event between the HA and the two nanoprobes takes place, resulting in the formation of a sandwich complex which subsequently brings the two nanoprobes closer together. This causes a decrease in QDs fluorescence intensity due to a non-radiative energy transfer from QDs to Au NPs. A resulting correlation between the targets HA concentrations and fluorescence changes can be observed. Furthermore, by utilizing the specific interaction between HA and glycan with sialic acid residues, the assay is able to distinguish HAs originated from viral subtypes H1 (human) and H5 (avian). The detection limits in solution are found to be low nanomolar and picomolar level for sensing H1-HA and H5-HA, respectively. Slight increase in assay sensitivity was found in terms of detection limit while exposing the assay in the HA spiked in human sera solution. We believe that the developed assay could serve as a feasible and sensitive diagnostic tool for influenza virus detection and discrimination, with further improvement on the architectures.

## 1. Introduction

Influenza, popularly known as “flu”, is one of the most common infectious diseases worldwide. The seasonal spreading of influenza (caused by both influenza virus type A and type B) results in 3 to 5 million cases of severe illness and about 250,000 to 500,000 deaths annually [1]. Avian Influenza A virus (AIV) is the only type associated with influenza pandemics and causes most of the deaths [2]. The subtypes of AIV are further classified according to the antigenicity of surface glycoproteins hemagglutinin (HA) and neuraminidase (NA). HA is the major protein on the viral surface [3]. Of the historical pandemics strains, subtype H1N1 (human-adapted), which caused the “Spanish flu” during 1918 to 1919, was the most virulent, causing 20 to 40 million deaths [4,5]. In 2009, a new H1N1 strain circulated among humans. Also in recent years, the highly pathogenic H5N1 virus known as “bird flu” has caused illness and death in birds and sporadically in humans [6]. Preventing the spread of AIV infection requires effective surveillance, and the need for a rapid, specific, and highly sensitive detection method still exists. 

Laboratory diagnosis of influenza virus infection is typically based on the established so-called “gold standard” viral culture and real-time RT-PCR [7]. However, these methods are either time consuming or labor-intensive, and may require special training and facilities [8,9]. Other popular methods are antibody-based immunoassays such as conventional ELISA [7,10,11]. However, ELISA assay has a comparatively low sensitivity and specificity. Commercialized lateral flow (immuno)assay kits have also been used for rapid detecting influenza virus in field, while acquisition of more semi-quantitative result remains a challenge.

Biological assays based on viral surface antigens including hemagglutination-inhibition (HI) and complement fixation are also referenced for WHO global surveillance [12]. The development of these methods largely depends on the interaction of HA with sialic acid (glycan) residue from the host cell surface. Transmission of AIV infections relies on the binding of HA to its receptor with the sialic acid residues, preferentially α-2,6 and α-2,3 sialic acids on human and bird cells, respectively [13,14,15]. By taking advantage of the interaction, glycan have been used as probes to detect and discriminate different strains from human and avian influenza in conjunction with other robust techniques including Surface Plasmon Resonance (SPR) technology [16,17], field effect transistor (FET) [18], waveguide mode [19], and colorimetric assay [20]. However, there remains a need for a sensing technique with simple, rapid, sensitive, and in-field capabilities for point-of-care influenza detection. 

Recently, fluorescence resonance energy transfer (FRET) has proven to be a powerful and sensitive tool in measuring the small changes of biological molecule interaction. FRET is a process in which non-radiative energy is transferred from an excited luminescent donor to a proximal ground state luminescent acceptor, or quencher, typically 1 to 10 nm away [21]. Several groups have introduced FRET techniques to detect and discriminate influenza viral nucleic acid sequences based on DNA hybridization [22,23,24]. The major limitation of these assays, however, is that additional reverse transcription PCR may be required to transcribe the influenza viral RNA genome to its complementary DNA sequence. An alternative method is to develop two antibodies involved sandwich FRET assay. However, application of larger antibodies complexes could cause a decrease in FRET efficiency, due to relatively large size of sandwich complex formed by two antibodies (4 ≥ 14 nm) [25]. To this point, small molecules such as glycan can be used as a promising probe for FRET assay. 

In the past decade, nanoprobes based FRET have attracted numerous attentions in biosensing field. In particular, luminescent semiconductor quantum dots (QDs) have proven to be highly effective donors in FRET assays due to tunable and narrow emission spectra, large extinction, and excellent photo stability [26,27,28]. In addition, colloid gold nanoparticles (Au NPs) serve as excellent fluorescence quenchers for FRET assays because of their extraordinary molar extinction coefficients and broad energy absorption bandwidth in the visible range [29,30]. The unique chemical reactivity of gold allows Au NPs to selectively bind a wide range of biological ligands or molecules. Au NP-QD pair based FRET sensors has been successfully demonstrated in biosensing applications with high sensitivity and selectivity [31,32,33,34].

**Figure 1:**
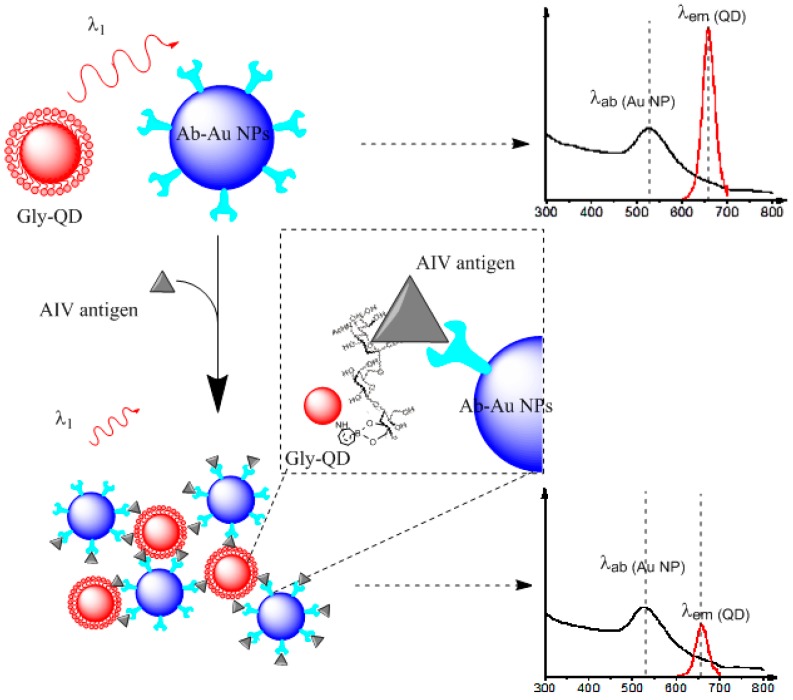
Schematic illustration of the assay architecture for sensing avian influenza virus (AIV) surface antigen HA.

Here, we propose a homogenous fluorescence-quenching assay for specific and sensitive detection and discrimination of influenza virus A HA antigen. The architecture of the assay includes a glycan-conjugated quantum dots (Gly-QDs) and another antibody-modified gold nanoparticles (Ab-Au NPs) pair. The mechanism of the assay is illustrated in Figure 1. Without HA antigen, no fluorescent quenching effect was observed; meanwhile, in the presence of HA, formation of Gly-QDs, HA, Ab-Au NPs sandwich complexes causes fluorescence quenching of QD fluorescence by Au NPs. The proposed assay was performed in a homogenous mode without additional washing steps and thus saves sensing time.

## 2. Experimental Section 

### 2.1. Reagents

Carboxyl quantum dots (QD655) were obtained from Life Technologies Inc. (Carlsbad, CA, USA). Recombinant influenza A virus H1N1 (A/New Caledonia/20/1999) HA, recombinant influenza A virus H5N1 (A/Viet Nam/1194/2004) HA, mouse monoclonal antibody to influenza A virus H1N1 HA (mAb-H1-HA), and mouse monoclonal antibody to influenza A virus H5N1 HA (mAb-H5-HA) were purchased from Sino Biological Inc. (Beijing, China). Two glycan, 6'-sialyllactose sodium salt (hereafter denoted as 6G) and 3'-sialyllactose sodium salt (hereafter denoted as 3G), were ordered from Carbosynth Limited Inc. (Berkshire, UK). All the other reagents were acquired from Sigma-Aldrich Chemical Co. (St. Louis, MO, USA). Only MilliQ water (18.2 MΩ) was used in the experiment.

### 2.2. Preparation of Antibody-Labeled Au NPs

Au NPs were prepared via the sodium citrate reduction of HAuCl4 as described by Frens [35] with slight modification. In brief, 100 mL of 1 mM HAuCl4·3H2O in water was heated until refluxing under stirring. Then, 5 mL of 38.8 mM sodium citrate tribasic dehydrate with water was quickly added to the solution under vigorous stirring. The mixture was then kept under refluxing for another 15 min. The color of the solution turned from yellow to clear, black, purple, and eventually to deep red. Afterward, the mixture was removed from heat and cooled to room temperature. The resultant solution was then filtered through a 0.45 μm acetate filter to remove large agglomerates. 

The citric-acid-capped Au NPs were then coated by monoclonal antibody (mAb) through well-established adsorption procedure [36]. Briefly, prior to coating, the pH of the Au NP solution (30 nM) was adjusted to 9.2 using 2 M NaOH. To determine the optimum concentrations of two antibodies to Au NPs, varying amounts of antibodies was added to 100 µL of pH adjusted Au NPs in order to get concentrations from 0.0 g/mL to 20 g/mL. The mixtures were then incubated for 30 min at room temperature under gentle shaking conditions. Finally, a NaCl solution (1 wt%) was added and mixed till the color change was completed. The optimum concentration is determined by observing the color of the solutions, as well as absorption spectra. Red-shift (the color becomes blue) indicates aggregation due to poor protection. Then, the pH-adjusted solution was incubated with an optimized concentration of mAb for 30 min at room temperature under gently shaking conditions. Next, bovine serum albumin (BSA) was added into the above solution to a final concentration of 1% (w/v) to passivate and stabilize the mAbs coated Au NPs. After 15 min incubation at room temperature under gentle shaking, the particles were purified by repeated centrifugation (12,000 g for 20 min at 4 °C). The pellet was re-dispersed in 10 mM phosphate buffer saline solution (PBS, pH 7.4) containing 1% BSA to obtain a solution of mAb-Au NPs with final concentration of 30 nM. The final solution was stored at 4 °C.

### 2.3. Preparation of Glycan-Functionalized Quantum Dots

Carboxyl-capped QDs were first modified with 3-aminophenyl boronic acid (APBA) through a carbodiimide reaction. In brief, a stock solution of carboxyl QDs (8 µM) was mixed with 10 mg/mL of APBA dispersed in PBS. After 3 min of gentle shaking, 10 mg/mL of N-ethyl-N′-(3 dimethylaminopropyl) carbodiimide hydrochloride (EDC) was added to the above solution. The solution was incubated at room temperature for 2 hrs under gentle shaking. Afterward, the resultant particles were purified through ultra-filtration (Amicon Ultra-0.5 mL centrifugal filters, 100 kDa MWCO, EMD Millipore Inc., Mississauga, ON, Canada). The molar ratios of QDs/APBA/EDC were maintained at 1/4000/6000. 

For glycan conjugation, the purified APBA-QDs were further mixed separately with each of the two different glycans using a feeding molar ratio of 1/6000 APBA-QDs/glycan for 30 min at room temperature. Each glycan conjugated APBA-QDs (Gly-QDs) solution was purified by ultra-filtration and resuspended in PBS to obtain a final concentration of 0.4 μM. The Gly-QDs solutions were stored at 4 °C under darkness for further use.

### 2.4. Characterization of Nanoparticles

Transmission electron microscopy (TEM) was used to characterize the morphology of the nanoparticles and the interaction of nanoparticles with or without antigen. The TEM images were obtained using a CM-12 microscope (Philips Electronic Instruments Inc., Mahwah, NJ, USA) operating at 120 KV. The bioconjugation of QDs by APBA and Glycan were verified using Fourier transform infrared (FTIR) spectrophotometer (Varian 660-IR, Agilent Technology Inc, Santa Clara, CA, USA). UV-visible absorption spectra were recorded by UV-3600 spectrophotometer (Shimadzu, Kyoto, Japan). The hydrodynamic diameters and size distribution nanoparticles were also measured in water by a Zetasizer Nano ZS Dynamic Light Scattering (DLS) system (Malvern, Worcestershire, UK).

### 2.5. Detection of AIV HA Antigen by Homogeneous Fret Assay

Detection of virus HA antigen was conducted in a black 96-well plate (Greiner Bio-One Inc., Vilvoorde, Belgium). The optimum ratio of Gly-QDs to Ab-Au NPs for the assay was firstly tested by adding various concentrations of Gly-QDs (from 1.5 nM to 37.5 nM) to the fixed concentration of Ab-Au NPs (10 nM) in a total assay volume of 100 μL in PBS buffer. In a typical assay, the detection of HAs was carried out by adding 10 μL of HA antigen sample solutions (with varying concentrations of HA from 38 pM to 60 μM) to 15 μL of an optimum ratio mixture of Gly-QDs and Ab-Au NPs. PBS was then added to each sample for a total volume of 100 μL. To evaluate the performance of our assay in complex matrix, 10 μL of human sera spiked with a known amount of HAs (38 pM to 60 μM) was added to the mixture of Gly-QDs and Ab-Au NPs in a final volume of 100 μL. In a typical experiment, the reaction plate was incubated at 37 °C for 1 hr under gentle shaking. The fluorescent intensity for each sample at λem = 655 nm was recorded by a Synergy H1 Hybrid Reader (BioTek Instruments Inc., Winooski, VT, USA) under λex = 470 nm. BSA (1% in PBS) was used as a negative control following the same procedure. All tests were conducted in triplicate. The quenching percentage (also known as quenching efficiency) was calculated as follows [37]:

$$Queching\;\left(\%\right)=\left(1-F/F_{0}\right)*\;100$$

where F and F_0_ are the mean Gly-QDs fluorescence intensities with and without quencher Ab-Au NPs, respectively. The maximum quenching efficiency was estimated at the saturation level of specific HA for each Ab-Au NPs/Gly-QDs system under optimum conditions.

## 3. Results and Discussion

### 3.1. Preparation of Gly-QDs and Ab-Au NPs

**Figure 2:**
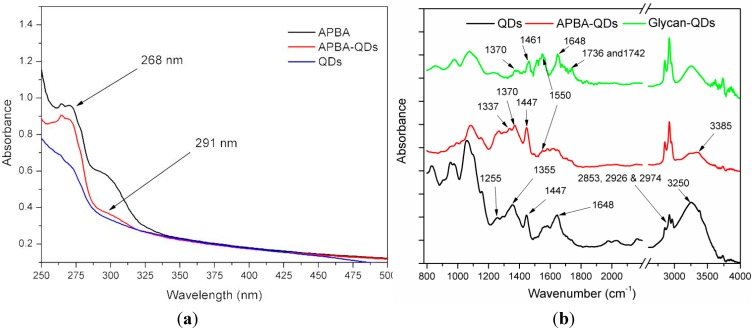
(**a**) Absorbance spectra of QDs with and without APBA conjugation; (**b**) FTIR spectra of bare carboxyl QDs, APBA-QDs and Glycan-QDs (6G).

Two glycans, 6G and 3G, were immobilized to QDs through APBA linkage to prepare the nanoprobes 6G-QDs and 3G-QDs, respectively. Initially, the carboxyl-group-capped QDs were conjugated with APBA through carbodiimide reaction. Conjugation of APBA to QDs was demonstrated (Figure 2a) by the new absorption peaks at 268 nm and 291 nm [38]. The phenylboronic acid groups from APBA are known to react with cis-1,2-diols to form cyclic esters at higher pH [39]. The conjugation of glycans was achieved by mixing glycans with the APBA-QDs. FTIR analysis was employed to confirm the bioconjugation of glycan to QDs. As shown in Figure 2b, in APBA-QDs, the new bands at 1330 and 1337 cm^−1^ confirms the conjugation of –APBA to QDs, which are attributed to the B-O stretching and C-B vibration, respectively [40]. In addition, the modes at 1550 cm^−1^ and 3385 cm^−1^ for N-H stretching are likely due to the conjugation amino groups from APBA to the carboxyl groups on the surface of QDs. The binding of glycan (sugar) to APBA-QDs can be confirmed by the presence of IR marker stretches at 1736 and 1742 cm^−1^ [40]. Detailed assignments for all FTIR spectra are listed in Table S1. 

To estimate the number of glycan molecules per QD, we tested the conjugation of APBA-QDs with another molecule exhibiting fluorescence property, nicotinamide adenine dinucleotide (NADH), using the same procedure. As shown in Figure S1, we could identify approximately 1096 NADH molecules per dot. It is anticipated that a similar number of glycan could thus be conjugated due to the similar reaction process. No significant change was found in terms of QD fluorescent spectra (Figure S2). Au NPs with average diameter around ~13 nm were used to absorb antibodies (Figure S3a). As protein concentration increases, a decrease in the absorbance at 580 nm occurs, which indicates that Au NPs become stabilized by antibody and less coagulated in salt solution [36]. The optimum concentrations of mAb-H1-HA and mAb-H5-HA to Au NPs solution (30 nM) were determined to be 20 μg/mL and 10 μg/mL, respectively (Figure S4a,b). A slight red-shift (2 nm from 524 nm to 526 nm) in maximum absorption was observed for the antibody well stabilized Au NPs, which could be due to the interaction of protein to the surface of Au NPs [36].

### 3.2. Detection of Influenza Virus HAs

**Figure 3:**
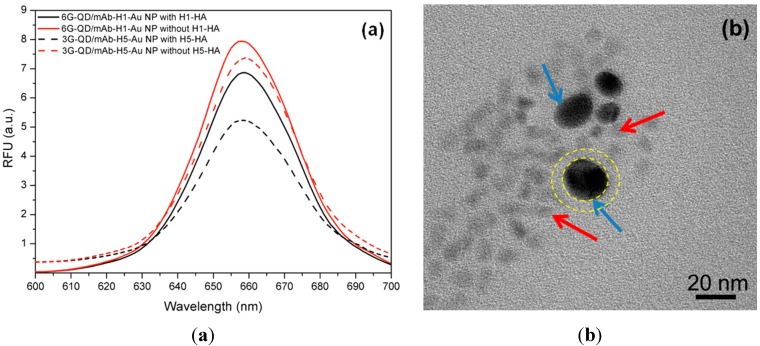
(**a**) Fluorescent spectra of 6G-QDs/mAb-H1-Au NPs mixture with and without antigen H1N1-HA; (**b**) TEM image of 6G-QDs/mAb-H1-Au NPs mixture after incubation with H1N1- HA. Blue arrow indicates Ab-Au NPs which has higher electron density, while red arrow indicates Gly-QDs which has a relative lower electron density.

The response of the assay to various concentrations of HAs is shown in Figure 3. As expected, addition of specific HA to selected mixture of Gly-QDs and Ab-Au NPs results in decreasing QD fluorescent intensity (Figure 3a), which indicates the binding of antigen by the two nanoprobes and thus formation of sandwich aggregates. Aggregates formed in high HA concentrations was observed. TEM image further demonstrates the aggregation formed between two nanoprobes in the presence of specific HA (Figure 3b). No aggregate was found for the control group without HA antigen (Figure S3c). The aggregates thus bring Au NPs close to QDs and quench the fluorescence in a non-radiative energy transfer mode [41]. Similar results were also found in the assay for sensing H5N1-HA (Data not shown).

The condition of the assay was further optimized. We selected the molar ratio of Ab-Au NPs to Gly-QDs to 0.15:1 for our assay (Figure S5a) to save Ab-Au NPs materials. We further tested the incubation time for the reaction. Figure S5b shows that significant fluorescence quenching was found after 20 min of incubation. 

**Figure 4:**
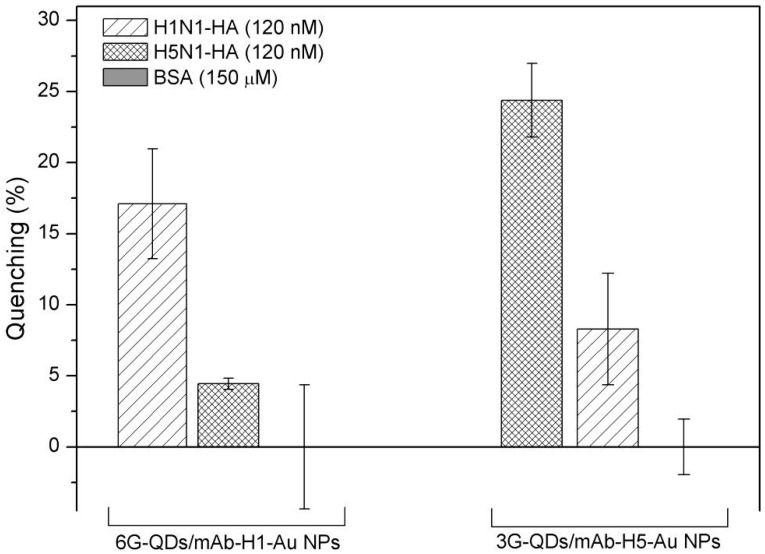
Cross-reactivity of two assays with human influenza virus H1N1 HA and avian influenza virus H5N1-HA. The mixtures, prepared with 6G-QDs/mAb-H1-Au NPs and with 3GQDs/mAb-H5-Au NPs, were tested using HAs derived from A/New Caledonia/20/1999 and A/Viet Nam/1194/2004 (120 nM), respectively. The control experiment was carried out using BSA (150 μM), (N = 3).

We then evaluated the cross-reactivity of our assay in the presence of HAs from two virus strains, namely, human H1N1 and the avian H5N1. For the 6G-QDs/mAb-H1-Au NPs mixture, binding of human H1N1-HA resulted in significant fluorescent quenching (Figure 4). Very low quenching effect was found upon addition of H5N1-HA to 6G-QDs/mAb-H1-Au NPs mixture. This low quenching effect could be attributed to non-specific interaction between 6G glycan (and mAb-H1) and H5N1-HA. Similar results were found in the mixture of 3G-QDs/mAb-H5-Au NPs, as shown in Figure 4. No significant quenching effect was found while adding BSA in either mixture. The results suggest that the adsorption of HAs was specific against the selected pair of nanoprobes, Gly-QDs and Ab-Au NPs. Thus, it is indicated that the immobilized glycan on the QDs maintained their original function as the receptor ligand for specific influenza virus HA. It is assumed that selection of highly specific mAb could eliminate the false signal. Detection limit was then evaluated under optimized reaction conditions. Specifically, incubation time was set up to 1 hr to ensure enough time for interaction of antibodies with HAs.

**Figure 5:**
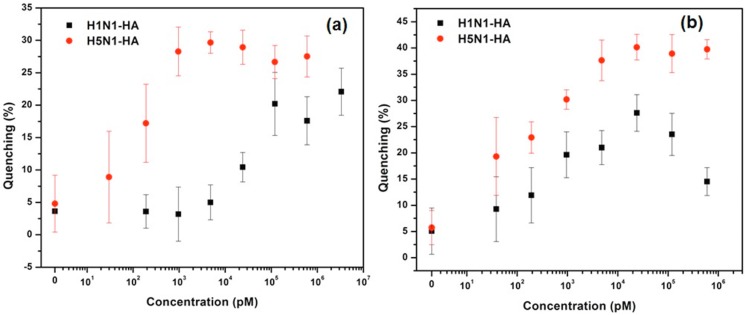
Detection of human influenza virus H1N1-HA and avian H5N1-HA by 6G-QDs/mAb- H1-Au NPs mixture and 3G-QDs/mAb-H5-Au NPs mixture, in PBS (**a**) and in human sera spiked PBS (**b**), respectively. (N = 3).

Concentration curves (Figure 5a) were obtained by adding various concentrations of HAs to the assigned Gly-QDs/Ab-Au NPs mixtures over the three experimental repetitions. Both assays show a characteristic linear trend with increasing assigned HA concentration until a saturation region is reached at high HA concentrations. The saturation regions were from 120 nM to 3.3 μM for H1N1-HA assay and from 4.8 nM to 600 nM for H5N1-HA assay. R^2^ values of 0.8339 and 0.9319 were achieved for the linear region. The detectable HAs concentrations could be as low as to 12 nM and 38 pM, comparing the average quenching percentages to those of negative control, respectively. However, due to relative large variation, the detection limits were secured to 60 nM and 190 pM for H1N1-HA and H5N1-HA, respectively, by using the current resolution of the plate reader combined with the standard deviation of the concentration curve when no HA were presented.

The relative large variation in in our current assay could be attributed to uneven distributions of nanoprobe FRET pair in the microplate wells. Manually mixing and transportation of the reagents (Gly-QDs, Ab-Au NPs, analyte) to microplate wells in our current assay mode might contribute this uneven distribution. Future automation and optimization of the assay should lead to an even greater degree of reproducibility. The sensitivity of our current assay format was found comparable with or even higher than those obtained in some recently developed influenza antigen HA biosensors (Table 1).

**Table 1:** Comparison of the sensitivity of the developed biosensors for the detection of influenza virus.

Sensor	Probe	HA Derived Influenza Type	Sensitivity or Affinity	Remark	Reference
SPR	Glycan-ligands	H1N1	K_d_ 1.5 µM (10 µg/mL, ~17 µM)	Low affinity and non-specific interaction.	[17]
QCM	Glycan	H5N3	K_d_ 14.4 nM	Good affinity.	[42]
SPR	Glycan	H5N1	K_d_ 1.6 nM	High affinity; specific substrate (Biacore chip) required.	[43]
Waveguide	Antibody	H1N1, H5N1	1 nM	Dissociation of dye from antibody may decrease sensitivity.	[44]
SPR	DNA Aptamer	H5N1	K_d_ 4.65 nM	Good affinity.	[45]
Electrochemical	Immuniliposome-Ru	H1N1	3 × 10^−14^ g/mL (12 fM)	Very high sensitivity; complex probe preparation step.	[46]
SPR	RNA Aptamer	H3N2	K_d_ 120 pM	High affinity; unstable RNA aptamer probe.	[47]
Interferometry	RNA Aptamer	H3N2	10 nM	RNA aptamer; relative low sensitivity.	[48]
SPR	RNA Aptamer	H1N1	K_d_ 67 fM	High affinity.	[49]
Field Effect Transistor (FET)	Glycan	H5N1, H1N1	50 aM	Very high sensitivity.	[50]

To further elucidate the analytical reliability and applicable potential of the developed assay, serial diluted HAs were spiked into human sera samples for the testing. Figure 5b (dark dots) shows similar result for H5N1-HA assay in human sera solution (Figure 5b, red dots) in terms of assay linear response region with R^2^ value of 0.9014 and secured detection limit at 38 pM. For H1N1-HA assay in sera sample, the linear response region was found similar to that of H5N1-HA in sera sample, with the R^2^ values of 0.9014. The detection limit was secured to 0.96 nM. Increasing of quenching percentage was observed in both assay formats in comparison to that obtained in PBS buffer. This could be attributed to the higher fluorescence intensity F0 in control groups, which is because the complex matrixes in sera diminish the nonspecific interaction between Ab-Au NPs to Glycan-QDs. From the definition of quenching percentage showed above, higher F0 may result in higher quenching percentage. This effect might also contribute to the relative higher sensitivity for both HAs in sera samples. Therefore, these results indicate that the developed quenching assay could be considered as a promising tool for rapid detection and identification of AIV-HA in clinical samples.

The stability of the assay was evaluated by running the tests on specific HAs samples (at 24 nM) with the reagents (Gly-QDs and Ab-Au NPs) stored for 5 months at 4 °C, respectively. Figure S6 shows that there is no significant change in fluorescence quenching percentage compared to the fresh prepared reagents for the same HAs concentration in Figure 5a. The result confirmed that the assay was still usable after 5-month storage at 4 °C.

It should be noted that in the current assay format, we were still using antibodies for the detection and discrimination of HAs from different virus sub-strains. Selection of high affinity mAb may further improve the sensitivity of the assay, as antibody affinity is significant in the performance of immunoassay [51]. This could possibly explain that the five-fold difference in detection limit between H1N1-HA and H5N1-HA is due to higher affinity from mAb-H5. Furthermore, as discussed above, antibodies occupy large spaces (4–10 nm), which may decrease the energy transfer efficiency and impair the assay sensitivity. This effect may contribute to the relatively low fluorescence quenching efficiency (percentage) in our current assay format with approximately 30% in the saturation HA concentration under optimized condition. The interparticle distance between Gly-QDs and Ab-Au NPs in the energy transfer complex can be estimated to be around 8 nm as shown in the TEM image (*i.e.*, the distance measured between two yellow circles as shown in Figure 3b). This space could be attributed to the interaction between HA antigen and mAb, where the space occupied by HA antigen (mono) is estimated to be ~3 nm according to the manufacturer’s information and the effective diameter of mAb is around 8 nm [52]. However, it should be noted that Fc chain mAb is flexible (e.g., it may bend), so it is possible for the size of the Ab-Antigen to be slightly smaller. Although the space is a bit larger than the efficient transfer distance in traditional FRET, it should be noted that the distance in the pair of Au NPs and QDs could be up to 70 nm, depending upon the size of metallic nanoparticle involved [37].

It is anticipated that by replacing antibodies with small molecules, such as specific DNA aptamers, the assay sensitivity could be significantly improved. It should also be emphasized that rather than using sophisticated instruments in biosensors (Table 1), our assay architecture will require only spectrometers, which are affordable in a general lab. In our current assay format, the reaction time could be as lower as 20 min. It is believed that miniaturization of the system on a microfluidic device could reduce the assay time by enhancing reaction efficiency [53]. Thus it is possible to incorporate our developed assay in a microfluidic platform for rapid, specific, and sensitive and field-deployable detection and discrimination of influenza.

## 4. Conclusions 

We have developed a simple, specific, and sensitive homogenous FRET assay to detect and discriminate human and avian influenza virus HAs (H1N1 and H5N1). The assay relies on the interaction between two nanoprobes: glycan (HA receptor sialic acid ligand)-immobilized QDs and HA-specific Ab-Au NPs. Upon addition of specific HA, sandwich hybridization occurs and triggers aggregation of the above two nanoprobes. As a result, QD fluorescence is quenched by the proximal Au NPs. The target HA concentrations have been correlated with the fluorescence change. Under optimum conditions, detection limits in the low nanomolar and picomolar range were achieved for H1N1-HA and H5N1-HA, respectively. Slight increasing in term of sensitivity was obtained in HAs spiked in human sera solution. The assay is highly selective toward HA of virus sub-strains from human and avian influenza viruses. To the best of our knowledge, it is the first time to use non-nucleic acid conjugated nanoparticles based FRET approach for influenza virus detection. In addition, the feasible way to conjugate QDs with glycan ligand will be of interest to other researchers to fabricate biosensors for influenza virus detection. With further improvement on the architectures, we believe the assay reported here is a promising tool to provide valuable information for the prevention of influenza pandemics.

## Supplementary Files

Supplementary File 1
