# Supplementary material for: A Homogenous Fluorescence Quenching Based Assay for Specific and Sensitive Detection of Influenza Virus A Hemagglutinin Antigen
Source: Sensors (Basel). 2015 Apr 15;15(4):8852–65. doi: 10.3390/s150408852 (PMC4431298; doi:10.3390/s150408852)
Supplement: Supplementary File 1 [file sensors-15-08852-s001.pdf]

OPEN ACCESS

sensors

ISSN 1424-8220

www.mdpi.com/journal/sensors

Supplementary Information

A Homogenous Fluorescence Quenching Based Assay for Specific and Sensitive Detection of Influenza virus A Hemagglutinin Antigen. *Sensors* 2015, *15*, 8852-8865

Longyan Chen and Suresh Neethirajan *

BioNano Laboratory, School of Engineering, University of Guelph, Guelph, ON N1G 2W1 Canada;
E-Mail: lchen266@uwo.ca

* Author to whom correspondence should be addressed; E-Mail: sneethir@uoguelph.ca;
Tel.: +1-519-824-4120 (ext. 53922); Fax: +1-519-836-0227.

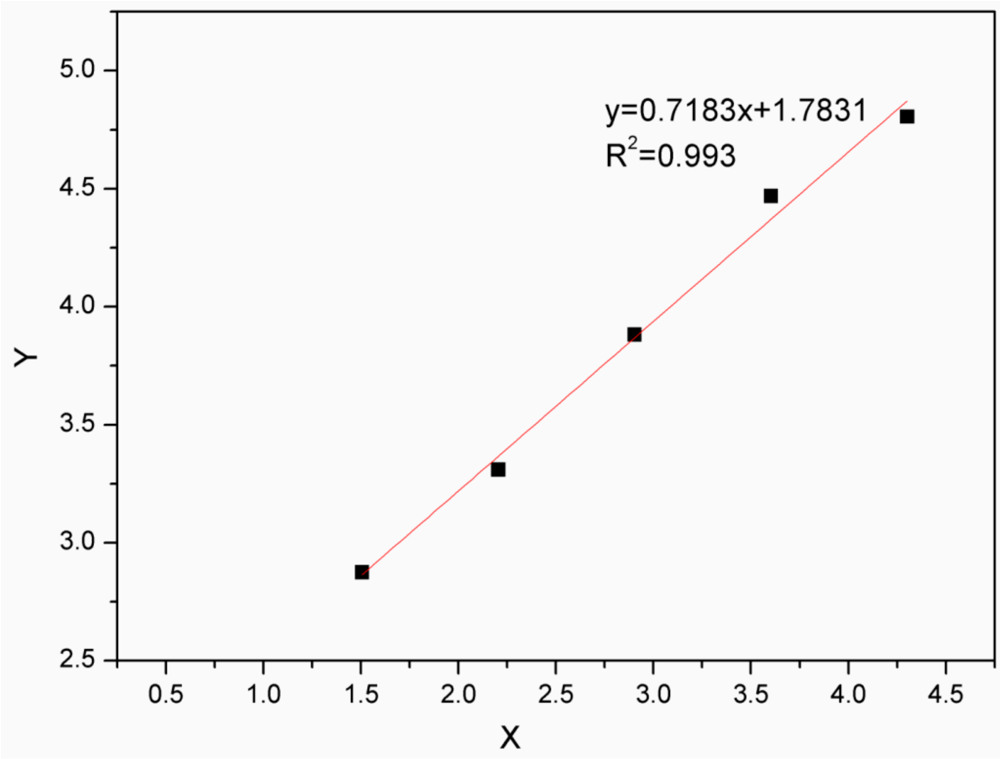

Figure S1. Determination of the number of NADH molecule per APBA-QDs, y = log (10,000× concentration of standard NADH solution in mg/mL), and x = fluorescent intensity of NADH at λ_{em} = 460 nm under excitation wavelength at 340 nm. The log number of average fluorescent intensity of NADH-QDs samples is 3.69 ± 0.02. The final average number obtained for NADH is 1906 ± 152 per QD.

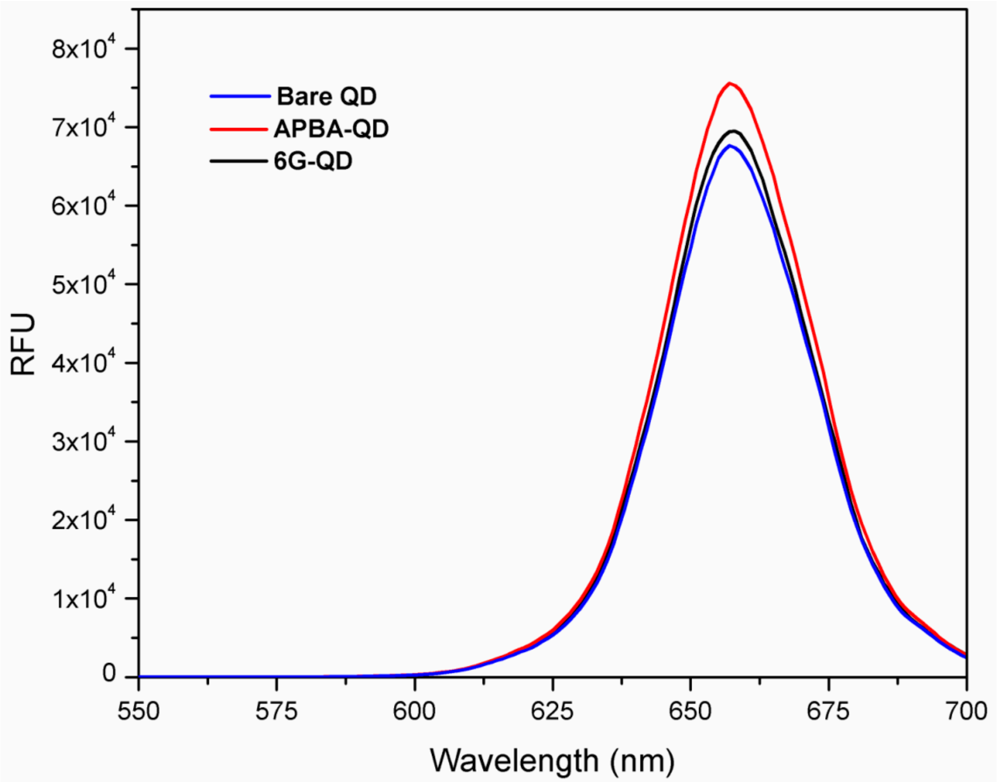

Figure S2. Fluorescent emission spectra of bare carboxyl QDs, APBA-QDs and glycan-QDs (6G) under excitation at 470 nm.

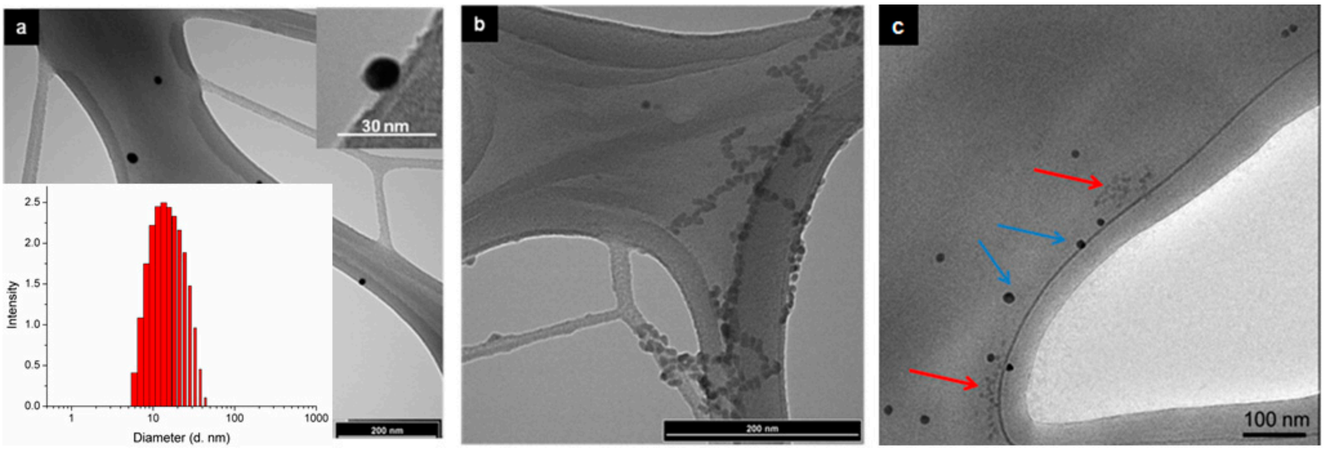

Figure S3. TEM images of (**a**) Ab-Au NPs (mAb-H1-Au NPs); (**b**) Gly-QDs (6G-QDs) and (**c**) the mixture of Gly-QDs (6G-QDs) and Ab-Au NPs (mAb-H1-Au NPs) without antigen H1HA.

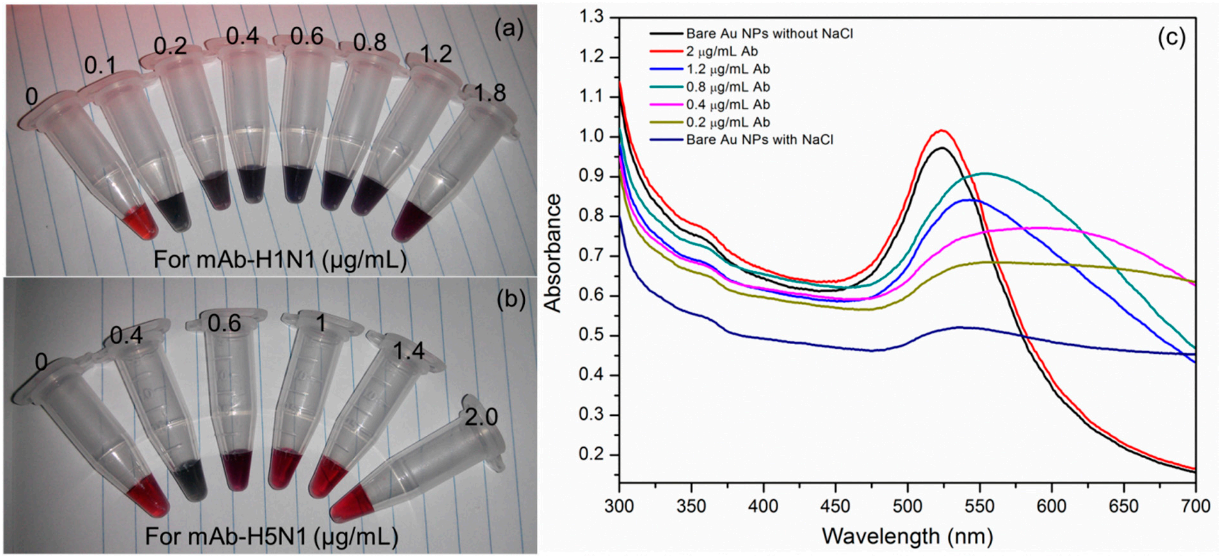

Figure S4. (**a**,**b**), testing of optimum concentrations of monoclonal antibody (Ab) for conjugation of gold nanoparticles (Au NPs); (**c**) UV-visible absorption of Au NPs and -Au NPs stabilized by different concentrations of anti-H1N1HA-mAb.

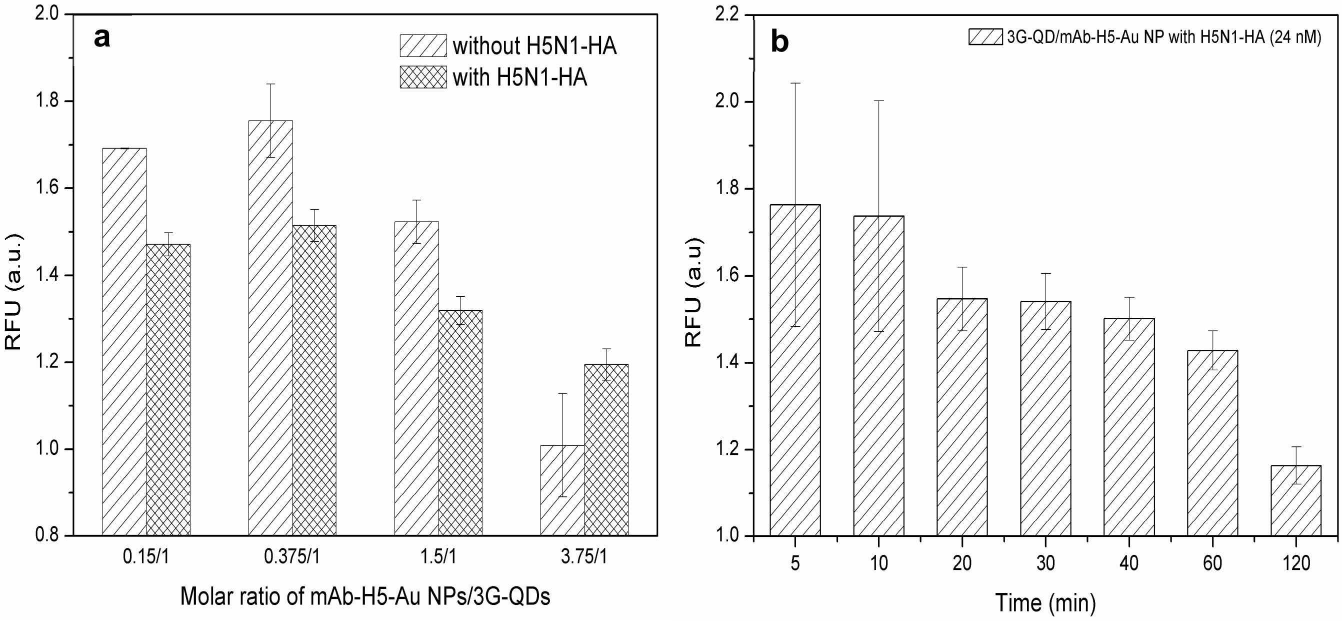

Figure S5. Optimizing fluorescent quenching based assay. Only the assay specific for H5N1-HA was evaluated. (**a**) Optimizing molar ratio of mAb-Au NPs to 3G-QDs in the presence or absence of H5N1-HA; (**b**) Optimizing assay incubation time. The concentration of H5N1-HA used in both tests is 24 nM.

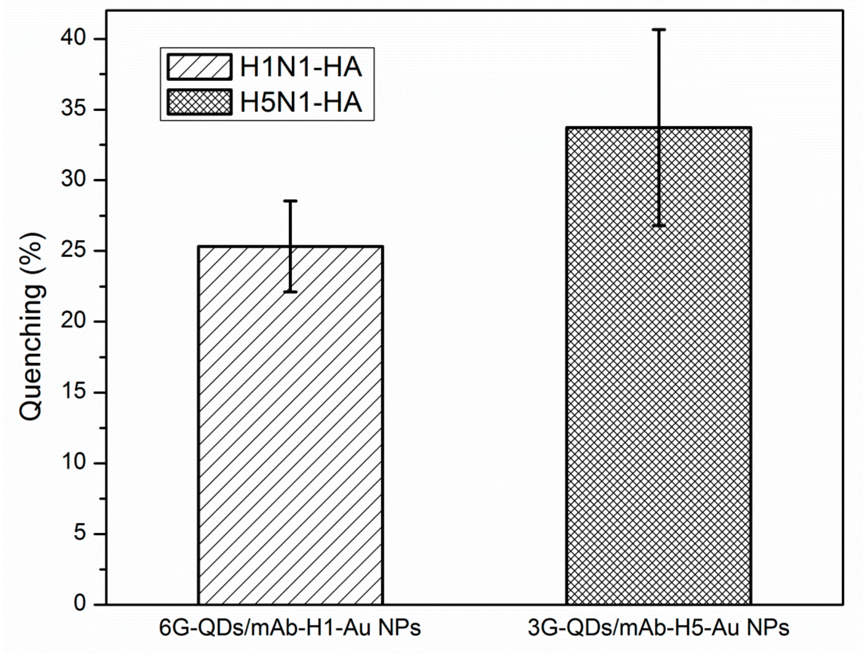

Figure S6. Assay stability test. The assay reagents pair (Gly-QDs and Ab-Au NPs) have been stored at 4 °C for 5 months. The test was performed by mixing the assay reagent pair with specific HA antigen (24 nM) under optimum condition. In the control group BSA (1%) was used to replace HA antigen. Quenching percentages were thus obtained by comparing the fluorescence signal from positive group (with HA) to that from BSA group as described in the method part of the manuscript.

Table S1. Assignment of vibrational frequency (cm^{-1}) in the FTIR spectra of QDs, APBA-QDs and Glycan-QDs.

Frequency, cm^{-1}	Assignment	References
1255	C-O-C vibration	[1]
1337	B-O stretching	[2,3]
1355	C-O stretching in carboxylic group	
1370	C-B vibration	[4]
1447	C-C stretching in phenyl group	[2]
1461	Undefined	
1550	N-H bending	[2]
1648	C=O stretching	[5]
1736, 1742	IR marker mode for phenylboronate ester formation	[2]
2846	Symmetric $-CH_2$	[2]
2916	$-CH_2$ vibration	[6]
2974	Asymmetric $-CH_2$	[2]
3250	-O-H stretching	[2]
3385	N-H stretching	[7]

References

1. Hofmann, A.; Thierbach, S.; Semisch, A.; Hartwig, A.; Taupitz, M.; Rühla, E.; Graf, C. Highly monodisperse water-dispersable iron oxide nanoparticles for biomedical applications. *J. Mater. Chem.* **2010**, *20*, 7842–7853.
2. Brewer, S.H.; Allen, A.M.; Lappi, S.E.; Chasse, T.L.; Briggman, K.A.; Gorman, C.B.; Franzen, S. Infrared Detection of a Phenylboronic Acid Terminated Alkane Thiol Monolayer on Gold Surfaces. *Langmuir* **2004**, *20*, 5512–5520.
3. Wang, H.; Liu, M.; Hu, X.; Li, M.; Xiong, X. Electrochemical Determination of Glycoalkaloids Using a Carbon Nanotubes-Phenylboronic Acid Modified Glassy Carbon Electrode. *Sensors* **2013**, *13*, 16234–16244.
4. Zhou, W.; Yao, N.; Yao, G.; Deng, C.; Zhang, X.; Yang, P. Facile synthesis of aminophenylboronic acid-functionalized magnetic nanoparticles for selective separation of glycopeptides and glycoproteins. *Chem. Commun.* **2008**, 5577–5579.
5. Tang, Z.; Guan, Y.; Zhang, Y. Contraction-type glucose-sensitive microgel functionalized with a 2-substituted phenylboronic acid ligand. *Polym. Chem.* **2014**, *5*, 1782–1790.
6. Liu, S.; Pang, S.; Huang, H.; Su, X. 3-Aminophenylboronic acid-functionalized $CuInS_2$ quantum dots as a near-infrared fluorescence probe for the detection of dicyandiamide. *Analyst* **2014**, *139*, 5852–5857.
7. Zhang, W.; He, X.; Yang, Y.; Li, W.; Zhang, Y. Selective capture and fluorescent quantification of glycoproteins using aminophenylboronic acid functionalized mesoporous silica coated CdTe quantum dots. *J. Mater. Chem. B* **2013**, *1*, 347–352.

© 2015 by the authors; licensee MDPI, Basel, Switzerland. This article is an open access article distributed under the terms and conditions of the Creative Commons Attribution license (http://creativecommons.org/licenses/by/4.0/).
